# Reducing Dietary Protein Levels with Amino Acid Supplementation in Duroc Sire Line Finishing Pigs: Growth Performances, Carcass and Meat Traits and Nitrogen Balance in Males and Females

**DOI:** 10.3390/ani14243572

**Published:** 2024-12-11

**Authors:** André Martinho de Almeida, María Angeles Latorre, Guillermo Ripoll, Marçal Verdú, Javier Alvarez-Rodriguez

**Affiliations:** 1 LEAF—Linking Landscape, Environment, Agriculture and Food Research Center, Associated Laboratory TERRA, Instituto Superior de Agronomia, Universidade de Lisboa, Tapada da Ajuda, 1349-017 Lisboa, Portugal; 2 Departamento de Producción Animal y Ciencia de los Alimentos, Facultad de Veterinaria, Universidad de Zaragoza-IA2, Calle Miguel Servet 177, 50013 Zaragoza, Spain; malatorr@unizar.es; 3 Centro de Investigación y Tecnología Agroalimentaria de Aragón (CITA)-IA2, Unidad de Producción y Sanidad Animal, Avda. Montañana, 930, 50059 Zaragoza, Spain; gripoll@cita-aragon.es; 4 Department of Animal Nutrition and Feed Industry, BonÀrea Agrupa, 25210 Guissona, Spain; marsal.verdu@bonarea.com; 5 Departamento de Producción Animal y Ciencia de los Alimentos, Escuela Politécnica Superior, Universidad de Zaragoza, Carretera de Cuarte s/n, 22071 Huesca, Spain

**Keywords:** amino acid supplementation, ideal protein concept, reduced protein content, sex

## Abstract

Crude protein (CP) reduction and amino acid (AAs) supplementation is an interesting cost-reducing strategy for environmental compliance. The objective of this work is to study productive performances of heavy Duroc X (Landrace X Large white) male and female pigs during the finishing stage under a 2% CP reduction with crystalline AA supplementation. A total of 60 entire male and 60 female pigs (means ± standard deviations: 109 ± 4.9 days of age; 67.9 ± 7.18 kg of body weight) were distributed in split-sex pens (6 pigs/pen) and fed ad libitum. Two isoenergetic diets were provided (145 and 125 g CP/kg with AA supplementation, control and low-CP) during the final 42 days of finishing period. Growth performance, carcass and meat traits were evaluated. The fixed effects of dietary treatment, sex and their interaction were considered and simple least squares models used. Males were 7.25% heavier (*p* < 0.001) and had 16% higher average daily gains (*p* < 0.0001) than females in both treatments with no differences in feed intake or feed conversion ratio (*p* > 0.05). Nitrogen efficiency was 35% higher in low-CP males. Little difference was found for most carcass and meat traits between treatments. Males had 7–8% higher loin subcutaneous fat thickness in both dietary treatments (*p* = 0.011). Males and females of the low-CP diet showed higher fat and saturated fat contents than controls (*p* = 0.020). A 20 g/kg CP reduction with AA supplementation is an efficient strategy during the finishing phase without productivity losses in heavy Duroc sire line genotypes of both sexes.

## Simple Summary

Crude protein (CP) content reduction with amino acid (AA) supplementation in the diet of pigs is an interesting cost-reducing strategy with benefits to the environment. The subject has been studied in light genotypes, particularly during the post-weaning and growth stages, with a limited number of studies addressing heavy Duroc sire line genotypes of both sexes. We hypothesized that entire males and females will be affected similarly when submitted to such a strategy. We conducted a study with sixty male and sixty female finishing pigs subjected to two isoenergetic diets differing in CP content (145 and 125 g CP/kg with AA supplementation, control and low-CP). We evaluated growth performance, carcass and meat quality traits. Males had similar performances in both dietary treatments and had higher weights and average daily gains than females. Carcass and meat traits were similar across sexes. Animals in the low-CP diet had higher fat and saturated fat contents than controls. This strategy can be used in heavy Duroc sire line genotypes of both sexes in the finishing phase.

## 1. Introduction

The population of the world is expected to grow to over 10 billion in the next two decades [1]. Population, urbanization and income growths will ultimately result in a rise in the demand for food of animal origin, particularly in developing countries. Therefore, the production of corn and soybean meal (SBM), two major feedstuffs also used in human diet, will also have to increase. This will lead to economic and environmental sustainability concerns, particularly with issues such as feedstuffs costs and availability, land use, natural environment degradation and greenhouse gas (GHG) emissions [2]. Meat products are of the utmost importance to human diets providing high value nutrients, in particular crude protein (CP), amino acids (AA), lipids, vitamins and minerals [3]. Pork production is thus crucial to answer these questions and feed a growing human population [4].

According to van Wagenberg et al. [5], nutrition represents over 70% of pork production costs. This is particularly important in high-production cost and feedstuff shortages scenarios, particularly the two main components of monogastric feeds: cereals and SBM. This aspect is coupled to the need to curb GHG emissions, effluent outputs and improving animal welfare. Protein and AA are among the priciest components in swine nutrition. Indeed, prices of SBM and its current price volatility represent a significant percentage of feeding costs affecting the pork production sector and ultimately consumer prices [5]. Reducing protein-rich feedstuffs costs in swine diets is thus of the utmost relevance.

One of the most proven strategies to achieve these goals is to reduce dietary crude protein coupled to in-feed AA supplementation. This is possible as dietary AA requirements can be matched without excessive dietary CP by means of the availability of crystalline AA, with particular relevance to lysine, methionine, threonine, tryptophan, valine, arginine, isoleucine and histidine [6,7,8,9,10]. This strategy has been studied for instance in piglets [6,7], growers [8,9] and finishers [10] of different genetic lines with varying results that range from decreased to similar production performances of animals fed below CP recommendations when compared to controls. Indeed, as the as the performances are maintained, it could be argued that current recommendations clearly surpass the real AA requirements. This strategy is nonetheless particularly troublesome for piglets or growers of high-growth lean genetic lines that have very high CP and AA requirements. Another strategy, made possible when the costs of synthetic AA are decreased, is to compensate the CP decrease by balancing the contents of essential AA towards an ideal protein concept [11,12,13,14,15]. Such a strategy has been applied in piglets, growers and finishers. Different examples may be found in the literature for piglets [11], growers [12] and finishers [13], as well as for breeding boars [14] or lactating sows [15]. Overall, the strategy is very interesting in efficiently reducing feeding costs without decreases in growth, carcass and meat productive performances. However, the response on productive parameters may be dependent on the genetic type and sex because of divergent lean growth requirements. Nevertheless, this strategy has additional environmental benefits by decreasing ammonia and GHG emissions in pig slurry production and storage [16], ultimately reducing acidification, eutrophication and odor impacts of pig manure [17].

Modern pig production systems, particularly those using lean Pietrain X (Landrace X Large white) crosses since the 1960s and the unsuitability of such genotypes for the production of traditional hams and sausages led to a pork production sector based on Duroc crosses. These include crosses with Iberian breeds [18] or with Landrace X Large white commercial dam lines [19]. Duroc crosses are often grown to heavier weights than other lean genotypes (i.e., Pietrain sired) without negative effects on productive performance and efficiency, ultimately leading to heavier and fattier carcasses that are suitable for both the specialized upscale traditional pork product markets and the standard pork meat market.

The reduction of dietary CP contents, with or without AA supplementation, has been studied in lean genotypes, in particular using Pietrain X (Landrace X Large white) crosses. Nevertheless, several studies may be found in the literature concerning CP reduction and AA supplementation in Duroc X (Landrace X Large white) crosses [20]. These are mainly focused on younger and lighter animals typically grown to standard commercial weights below 100 kg. Very few studies address older, heavier animals, which are additionally entire. Regardless of the genotype used, and to the best of our knowledge, no studies have focused on the effects of CP reduction and AA supplementation on entire male or female finishing pigs at weights above 100 kg of live weight. This is, however, a very pertinent subject. Indeed, albeit nutritional recommendations for growing and finishing pigs are often the same for both males and females [21], the two sexes have different nutritional needs and will ultimately be differently affected by CP reduction and AA supplementation.

The objective of this work was to study productive performances of heavy Duroc X (Landrace X Large white) male and female pigs during the finishing stage under a 2% CP reduction with crystalline AA supplementation. It was hypothesized that males and females will be equally affected by the CP reduction with AA supplementation strategy and that animals fed reduced CP with AA supplementation will have a productive performance similar to those of control animals.

## 2. Materials and Methods

### 2.1. Animals and Experimental Design

This study was performed according to the guidelines of the Spanish Animal Protection Regulations RD 53/2013, which complies with the European Union Directive 2010/63. Additionally, an in-house Animal Experimentation Committee (University of Lleida code CEEA 01/23, 25 January 2023) supervised the animal experimental protocols.

A total of 120 pigs, 60 intact males and 60 females, originated by Duroc sires x (Landrace x Large White) dams were used. At 9 weeks of age, the animals were allotted in split-sex pens (6 pigs/pen, 0.74 m^2^/animal) with slatted floor, cup-drinker and an individual single-space feeder with dry feeding, in the experimental facilities of Nial, part of the bonÀrea Agrupa company (Guissona, Spain). The barn had natural ventilation with lateral windows directing the air regulated through temperature sensors (1.5 m ground height). The average air temperature throughout this study was 21.4 ± 2.20 °C and relative humidity was 55.9 ± 7.79%. The experimental diets were supplied during the last 6 weeks of the finishing period, from 15 weeks of age, starting with an initial body weight (BW) of 67.9 ± 1.75 kg.

Two diets with different target dietary CP levels were provided (145 and 125 g CP/kg). Table 1 shows the composition in ingredients and estimated nutrients. These diets were formulated to meet the ideal protein concept and adapted to finishing pigs using the FEDNA nutrient recommendations [21]. In the low-CP diet, the reduction of protein sources was mainly achieved by replacing barley and wheat middling, and crystalline AAs were added to avoid essential AA deficiencies. Both diets were formulated to be isoenergetic (10.2 MJ Net Energy/kg) and to have a similar dietary electrolyte balance (Na^+^ + Cl^−^ + K^+^ = 155 mEq/kg). Each experimental diet was replicated five times (five pens per diet and sex). Water and feed were provided ad libitum, the latter in pelleted form. The trial lasted 42 days, after which the animals were slaughtered.

Feed samples were collected before starting the trial to analyze basic chemical composition. The analyses were carried out in duplicate, following the procedures of the AOAC (2005) [22]: dry matter (DM) by oven drying method (934.01), total ash by muffle furnace (942.05) and CP by Kjeldahl method (976.05) using a Kjeltec^TM^ 2300 analyzer (FOSS analytical, Hilleroed, Denmark ). The AA composition was analyzed in an external laboratory (Office, Barcelona, Spain) by HPLC-Fluorescence (norms UNE-EN ISO 13903 [23] for all amino acids except tryptophan and UNE-EN ISO 13904 for tryptophan [24]). The results for nutrient composition are shown in Table 1.

### 2.2. Growth Performances and Experimental Sampling

During the trial, supplied water and feed were recorded daily, whereas animals were weighed on days 0, 21 and 42 using a digital scale, thus allowing the calculation of ADFI (average daily feed intake), ADG (average daily weight gain), FCR (feed conversion ratio), water use and water to feed ratio, on a pen basis. To analyze fecal moisture content, feces were sampled on days 21 and 42 by direct rectal stimulation of three pigs per pen and subsequently pooled (300 g/pen, approximately). Pig nitrogen (N) excretion was calculated as N intake (a function of feed intake and feed N content) minus N retention (a function of CP content (15.3% in females, and 16.1% in males, according to [21] and N content (16%) in BW gain). The final N content that remained in pig slurry was estimated using a standard N volatilization rate of 28.75%, based on regional legislation compliance standards [25].

### 2.3. Carcass and Meat Traits

At the end of the trial (42 days), animals were slaughtered in a commercial abattoir (bonÀrea Agrupa, La Closa, Guissona, Spain) using standard commercial practices [26]. Briefly, pigs spent a 16 h lairage period with access to water but not feed. Animals were stunned with 85% CO_2_ for 120 s and immediately exsanguinated, scalded, de-haired, eviscerated and halved down the midline. Each hot carcass was weighed and dressing percentage determined. Ham fat thickness and lean meat percentage were obtained with an AutoFom III ultrasonic system (Frontmatec A/C; Herlev, Denmark). Boar taint was monitored by human nose scoring [27]. This method classifies carcasses as tainted and non-tainted by a trained assessor that heats fat tissue directly on-line. Carcasses were then refrigerated at 2 °C (1 m/s of air speed; 90% of relative humidity) for 72 h before joint cutting.

A longissimus lumborum sample (approximately 300 g) was taken from the caudal part of each left half carcass and vacuum-packaged in plastic bags before freezing at −20 °C until subsequent analyses. The samples were thawed for 24 h at 4 °C, removed from the packages, blotted dry for 15 min, and weighed. Thawing losses were calculated by dividing the difference in weight between the fresh and thawed samples by the initial fresh weight. Thawed samples of the loins were used for pH and color measurements.

Color attributes in the CIELab space were measured with a chromameter (Konica Minolta CM 2002, Osaka, Japan) as described [28]. Briefly, D65 was the illuminant used and 2° was the standard observer position. The chromameter had been previously calibrated with a blank following instruction from the manufacturer. Averages of three readings were used to determine lightness (L*—greater values indicating lighter color), redness index (a*—greater values indicating redder color) and yellowness index (b*—greater values indicating more intense yellow color). Chroma (C*) and hue angle (*h_ab_*) were calculated as described [28]. Chroma is an indication of the quantity of pigments, with higher values indicating a more vivid color, lacking in greyness and hue being the attribute of color perception denoted by blue, green, yellow, red, purple and so on related to the pigment state. The pH was measured using a portable pH meter (Crison Instruments, Barcelona, Spain) equipped with a glass electrode.

A Texture Profile Analysis (TPA) test was carried out in one randomly selected animal per pen. It was conducted on thawed samples using a 5543 Texturometer (Instron, Norwood, MA, USA) provided with a compression cell of 500 N load applied at a cross-head speed of 1 mm/s. The test consisted of two compression cycles at 50% of the original portion height with an aluminum cylinder probe with 60 mm of diameter. A time of five seconds was allowed to elapse between the two compression cycles. Raw meat samples were obtained with a circular puncher (25 mm of diameter) and tailored to a height of 20 mm (6 replicates per sample). The primary TPA characteristics were considered: hardness (maximum force of the first compression cycle required to compress the sample, in N), cohesiveness (extent to which the sample could be deformed prior to rupture, dimensionless) and springiness (ability of the sample to recover its original form after the deforming force was removed, dimensionless).

Chemical composition of meat (moisture, ash, protein, collagen, fat and saturated fat) was also obtained on the same randomly chosen animal per pen. It was determined with a FoodScan2™ meat analyzer (Foss Electric, Hillerød, Denmark) by near-infrared transmission spectroscopy with calibration using artificial neural networks and transmittance mode, according to AOAC method 2007.04 for meat [29]. Each sample was minced and put in a circular cup of 13.8 mm of depth and 140 mm of diameter that was introduced in the FoodScan™2 equipment (FOSS Iberia S.A., Barcelona, Spain).

### 2.4. Economic Assessments

The feed costs and carcass incomes were calculated for the best and least favorable scenarios in weekly prices during the last three years (the lowest feed and carcass prices were attained in 2020, before the Ukrainian conflict, while the highest favorable scenario was attained in 2022). The feed price of the control diet coincided with the mean values of finishing feed in Spain throughout these years. In all cases, feed cost was calculated by multiplying feed price (EUR/kg of feed) by the amount of feed consumed during the 42 days of experiment (in kg/pig) plus a partial fixed feed cost of EUR 80/pig from 6 to 70 kg of BW (start of the experiment). Carcass income (EUR/pig) was obtained by multiplying the carcass price (EUR/kg of carcass) by the carcass weight (kg of carcass). Margin-over-feed-cost (EUR/pig) was calculated by difference between carcass income and feed cost.

### 2.5. Statistical Analysis

Data were analyzed with the statistical package JMP Pro16 (SAS Institute Inc. Cary, NC, USA). Growth performances, nitrogen balance, water use, fecal consistency and carcass and meat quality variables were analyzed with simple least squares models that included the dietary treatment (control or low-CP), the sex (entire males and females) and their interaction as fixed effects. The experimental unit was considered the pen for growth performances and N balance (*n* = 5 replicates per dietary treatment and sex), whereas for carcass and meat quality variables it was considered the animal (*n* = 30 replicates for carcass and *n* = 5 replicates for meat, per dietary treatment and sex). Two male carcasses from the control treatment were excluded from the analysis as a result of an unintentional carcass line discarding. The results are expressed as least square means and their standard errors. The separation of means was carried out with Tukey’s test. The association between dietary CP level and sex with the superior carcass grade (>60% lean content) according to EU grading, as well as between dietary CP level and scoring for boar taint in entire males, was evaluated with Pearson’s test to a contingency table. The effect of boar taint incidence according to BW was evaluated by including the fixed effects of dietary CP level and boar taint presence on the slaughter BW. Significance was declared when *p* < 0.05, but tendencies were commented when the level of significance was in the 0.050.10 interval.

## 3. Results

Growth performances of male and female pigs under the tested diets are shown in Table 2. At the onset of the trial, animals had similar live weights of around 68 kg. However, at day 21, with animals weighing 90–95 kg, differences were noticeable, with males being 5–6% heavier than females although no significant differences were recorded regarding the diet factor. The same trend was recorded at day 42, with males being 7.2% and 7.3% heavier than females for the reduced CP and control dietary treatments, respectively. A similar pattern was recorded for ADG, with males having 16% higher ADG than females in both nutritional treatments. Regarding ADFI, no differences were recorded because of sex for both diets. Finally, for the three weighing periods considered, FCR was higher in females of both nutritional treatments with no differences being recorded regarding the latter factor.

Water use and water:feed ratios are shown in Table 3. From day 0 to day 42, the four experimental groups had water uses ranging from 8.1 to 9.9 L/pig/day with no significant differences being recorded. A similar trend was recorded for all measurement periods. In the same way, water:feed ratios ranged between 2.48 and 3.33 for the different measurement periods with no significant differences being recorded across them.

Fecal DM contents and nitrogen balance data are shown in Table 4. Fecal DM content was similar for the four experimental groups at day 21. At day 42, significant differences were recorded with low-CP males having lower fecal DM contents than the other three experimental groups. Regarding the N balance, significant differences were recorded for N consumed, with control animals consuming 17% more N than animals fed a low-CP diet. Regarding excreted N, it was significantly higher in control females by comparison to both control males and low-CP females. Finally, low-CP males had lower N excretions levels than the other three experimental groups. Retained N was 22% higher in males than in females for both nutritional treatments. Regarding N efficiency, it was higher in low-CP males by comparison to both low-CP females and control males, whereas control CP females had lower N efficiency than those of the other experimental groups.

**Figure 1:**
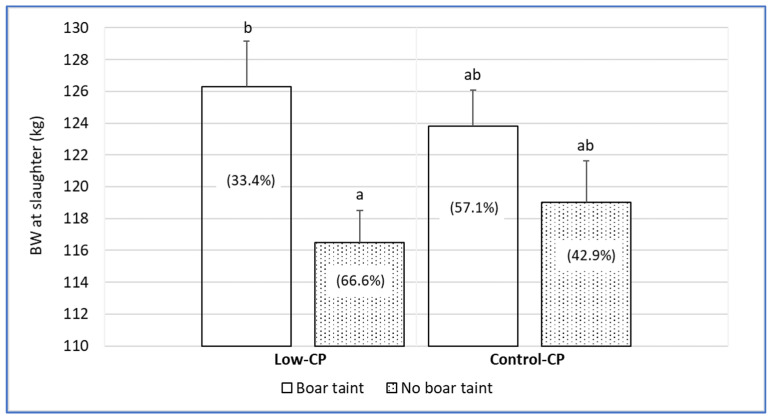
Body weight of entire male pigs in dietary treatments according to boar taint incidence (inside each bar, values between parenthesis indicate proportion of carcasses showing each condition in low-CP and control diets). ^a,b^ Bars with different superscripts indicate significant differences (*p* < 0.05).

Carcass traits are displayed in Table 5. Carcass weights were significantly higher in control (91.2 kg) and in low-CP (89.9 kg) males. The latter were in turn similar to carcass weights of females, irrespective of the nutritional treatment. Carcass yields were similar for the four experimental groups (75.1–75.8%), as were lean contents (60.0–60.9%) and minimal subcutaneous fat thickness of hams (12.0–12.7 mm). Regarding the subcutaneous fat thickness of loin, there was an effect of sex, with males having 7–8% higher values when compared to females in both dietary treatments. Low-CP females had a significantly higher percentage of carcasses of higher lean content (82.1%) by comparison to the three other groups that had values in the range 44.4–46.7% out of total carcasses of the group. Regarding boar taint in entire males (Figure 1), it did not differ across diets and it was detected in 33.4% and 42.9% in low-CP and control males, respectively. Boar taint in male pigs was more related with the BW at slaughter rather than with the diet.

Meat traits are shown in Table 6. The pH of meat after carcass cutting ranged between 5.24 and 5.28, being similar for all experimental groups. Similar trends were recorded for the different color traits ranging between 54.29 and 56.29 for lightness, 2.84 and 3.19 for redness, 9.48 and 10.64 for yellowness, 71.5 and 74.1 for Hue angle and 10.02 and 11.08 for Chroma. Regarding meat chemical analysis, moisture (71.53–72.70), ash (1.81–1.85), protein (25.68–26.38) and collagen (0.86–0.96) percentages were very similar across the four experimental groups. Regarding fat and saturated fat percentages, a strong dietary influence was noticeable with animals of both sexes in the low-CP diets having higher fat contents than animals in the control groups. Indeed, and regarding fat content, it was 42% higher in low-CP females when compared to control females, whereas in males it was 21% higher. Regarding saturated fat percentages, they were 65% higher in low-CP females when compared to control females, whereas in males it was 34% higher in the low-CP group. Finally, values for thawing losses (19.77–20.72%), hardness (29.7–39.9 N), cohesiveness (0.27–0.30) and springiness (3.47–4.90 cm) were statistically similar for the four experimental groups.

The economic assessment of this study is shown in Table 7. Under the two first scenarios considered (low feed and carcass prices and high feed and carcass prices), the decrease in CP content with AA supplementation leads to similar feed costs for all experimental groups: EUR 112.05–113.07/pig (scenario 1) and EUR 133.17–136.87/pig (scenario 2). Results are, however, different regarding carcass incomes and margin-over-feed costs. Indeed, in both scenarios, a strong influence of sex was recorded, with males having more interesting results for these two variables than females under the same nutritional treatment. A similar trend was recorded for the high feed and low carcass prices scenario. Regarding the low feed and high carcass prices scenario, control males had higher margins than low-CP females, whereas low-CP males and control females had intermediate values.

## 4. Discussion

At the beginning of the experiment, male and female pigs had very similar body weights as no significant differences were recorded at this stage. At day 21, however, differences between male and females became more evident, with males having higher BW than females. By day 42, males and females diverged significantly, with the former being significantly heavier than the latter. On the contrary, there was no dietary effect. A similar trend was recorded for ADG, highlighting differences between males and females. These results are expectable and are generally in accordance with studies conducted by researchers working with leaner genotypes than in the current study and fed reduced levels of CP and supplemented with crystalline AA [30,31,32]. They clearly indicate that the CP reduction level used in this study was adequately counterbalanced by the AA supplementation in both males and females without any major effects on the growth performance of the animals, other than sex differences between males and females. Interestingly, no differences were recorded for ADFI that were similar throughout the experiment for the four experimental groups. These results are in accordance with those of other researchers [13], albeit contrary to those found by Tuitoek et al. [33], whose performances were penalized when pigs were fed a lower dietary CP content (110 g/kg of feed from 55 to 100 of BW) than in the current experiment. Results indicate that animals of both sexes and dietary treatments consumed the same amount of feed during the experiment, in accordance with earlier sex comparisons in lean genotypes [34]. It could be speculated, however, that if the experiment would have been continued for a longer period, differences between males and females would eventually became noticeable. This would be a consequence of sexual divergence (6–7 months of age), albeit clearly extending the normal length of the finishing phase in commercial pig production.

The above-mentioned pattern of results led to interesting changes regarding FCR. Indeed, differences between males and females are noteworthy throughout the different phases of the experimental period with females having poorer FCR than males, as expectable and concurring with data by other researchers when comparing entire males and females. Indeed, Aymerich et al. [34] inferred that finishing lean entire males (70–110 kg) have a greater SID Lys:NE ratio requirement (ranging from 3.63–4.01 g SID Lys/Mcal NE) than females (3.10 g SID Lys/Mcal NE) to maximize growth performance and carcass leanness. When the dietary amino acid to energy ratio requirements are met, authors suggest that entire males display greater protein deposition, starting from 50–70 kg, when there were no evident differences in ADFI [34]. However, in the present study, the higher ADG of entire males was not coupled with greater carcass leanness than in females, which may indicate that the current entire males could respond to slightly higher SID amino acids to NE ratio supply. Accordingly, dietary AA supply in entire males may be tailored with net energy supply to surpass more than the current 3.55 g SID Lys/Mcal NE.

Regarding dietary effect, results show a clear dietary effect from day 0–21 (*p* = 0.02), with control animals having lower FCR than reduced CP animals. No dietary effect was found for the second period (day 21–42; *p* = 0.678) of the finishing period. Regarding the complete finishing period (day 0–42), animals had a pattern of results similar to that of the first period with an absence of interactions between the two studied factors. Overall, it can be inferred that regarding growth performances, both sexes reacted similarly to the reduced dietary CP reduction and that such reduction had little effects on BW gains, other than the expectable differences between males and females. It could also be inferred that nutritional recommendations used in commercial practices may be above the actual needs of animals in this particular physiological state. Regarding the efficiency of growth performances, males are, as expected, more efficient than females, regardless of the diet. Finally, differences in growth traits had no implications on water use and water:feed ratio. Indeed, no differences between groups were recorded throughout the experiment for both parameters. This result is coincident with previous experiments in which water intake and urinary output of pigs was unaffected by slight variations in protein content of diets [35,36].

Reflecting the differences between CP contents of the two dietary treatments, the results for N intake can be considered expectable. Indeed, controls of both sexes had higher levels of N intake when compared to pigs of both sexes in the reduced CP diet, similarly to the studies by Souza et al. [37]. Such results are not in line with daily N excretion. Indeed, for this parameter, sex and diet effects were noticeable, with males having lower N excretion levels than females in both dietary groups. Furthermore, control animals had in general higher levels of N excretion than animals fed with a reduced CP content, concurring with the results found by Shriver et al. [38].

Such a pattern of results leads to an independent sex and dietary influence on N efficiency, with controls and females being less efficient than animals fed with a reduced CP content than males. As expected, males are more efficient than females. These results for N efficiency point to a high interest in the reduced CP diet, when supplemented with AAs, in feeding of finishing pigs of both sexes. Indeed, the best N efficiency was achieved by males fed the low-CP diet, whereas males fed the control diet showed a similar N efficiency to females fed the low-CP diet. In any case, the worst N efficiency was displayed by females fed the control diet. This suggests that the adjustments in CP are especially recommended for this group. Fecal consistency was very similar for the four experimental groups, albeit low-CP Males had a lower DM content at day 42 in comparison to the other three experimental groups. The reasons for such differences are not entirely clear. However, they are likely linked to differences in N excretion that are lower in males fed with lower levels of dietary CP. It is plausible that differences in the intestinal microbiome of these animals that would ultimately lead to differences in digestion and absorption functions. Future studies on this issue should therefore be considered.

Carcass weights reflect the differences in body weights mentioned earlier. Indeed, there was a strong sex effect, with males having heavier carcasses than females, as observed, for instance, between castrated males and entire females [28], whereas dietary treatment led to no differences in carcass weights. The latter results are in accordance with other researchers [30,31], but are contrary to those found by Souza et al. [37]. Interestingly, and contrary to what would be expectable, no differences induced by sex or dietary treatment were recorded on carcass yield, lean content and minimal subcutaneous fat thickness of the ham. These results indicate that, similarly to BW measurements, a CP reduction with an appropriate AA balance will have little effect on pig carcass quality traits. The lack of differences could ultimately suggest that CP reduction is properly balanced by the AA supplementation. The only carcass characteristic where a treatment effect was noticeable was on the loin’s subcutaneous fat thickness that was higher in males than in females, similarly to the descriptions by Monteiro et al. [8]. Such differences are again an expectable consequence of the differences in body weight, but also a possible muscle tissue replacement with adipose tissue as a result of a mild restriction of the SID amino acid to net energy ratio requirement in males. Finally, results for boar taint detection point out an absence of differences between dietary treatments, albeit with heavier animals in both diets having a higher incidence of boar taint when compared to lighter animals. In accordance, it must be stated that according to the commercial practices conducted when using this genetic type, entire males must be slaughtered below 120 kg to assure low levels of boar taint in carcasses, regardless of dietary CP level.

Results for meat traits are very similar across the different treatments with parameters such as pH or color traits showing comparable values, regardless of sex and diet. Similarly, chemical composition also shows an absence of significant differences across the four experimental treatments, except for fat and saturated fat percentages that were higher in the meat of animals of both sexes fed the low-CP diet. As demonstrated, pigs with higher intramuscular fat tend to have also a higher content of saturated fat in their meat [39]. These results therefore suggest that, despite AA supplementation, the animals in the low-CP diet ceased their muscle growth at an earlier stage when compared to the control animals and started to deposit fat at a higher extent, leading to more saturated fat percentages. As for other meat traits, thawing losses, hardness, cohesiveness and springiness were also similar across the four experimental groups. These results point to a strong similarity of measured meat sensorial traits, irrespective of sex or dietary treatment, indicating that the CP reduction strategy balanced with AA supplementation leads to a meat with a consumer acceptance similar to that of the control animals. Nonetheless, acceptance has the potential to be higher as the palatability of pork longissimus dorsi is enhanced with intramuscular fat contents in the 3.5–4.5% range [40]. These results are consistent with others available in the literature [8,20]. It would be interesting, however, to conduct a sensory analysis with a trained panel to determine if such lack of differences is consistent when evaluated by humans.

Economic evaluations are not commonly conducted in the research available in the literature [41] as they are seldom considered. Herein, in order to evaluate the economic feasibility of the protein reduction strategy, we have conducted four distinct economic scenarios combining high and low feed prices to high and low carcass prices, taking into account the Spanish values for feedstuffs and carcass in 2020 and 2022. The combination of both high feed and high carcass prices led to margins-over-feed cost of about EUR 43.47–51.23. Under such a scenario, the only factor determining the margin value per pig is sex, with males having higher margins than females in both diets. Similarly, in the low feed and carcass prices scenario, the determinant factor in the establishment of margins per pig is again the sex of the animals, with values ranging from EUR 30–30.5/pig in females and EUR 37.30–39.57/pig in males. In both scenarios, results are a direct consequence of income associated to carcass and, in turn, a direct consequence of higher body weight and carcass weights attained by males, furthermore similar in both diets. On the contrary, feed prices that included feedstuffs such as cereals and soybean meal in addition to the crystalline amino acids show no differences in these two scenarios, with values ranging from EUR 112–113/pig in the low feed prices scenario and EUR 113.17–136.87/pig in the high feed prices scenario. It must be stated, however, that in the high feed and carcass prices scenario, there is a tendency towards higher feeding costs of EUR 2–3 per pig when the control diet is used. Regarding the two divergent scenarios (high feed and low carcass prices; low feed and high carcass prices), a similar trend is noticeable with sex being the only factor affecting margins per pig. Indeed, in the first of such scenarios, differences between male and female margins are particularly striking, with males practically doubling their margins when compared to females, regardless of diet. Accordingly, split-sex feeding during the finishing period is not often used in commercial pig productions systems around the world, but could be widely implemented to obtain economic and nitrogen use efficiency advantages when compared to mixed-sex feeding systems [41,42]. Finally, in the low feed and high carcass prices, the most favorable to the farmer, margins per pig are again higher in control males, reflecting the higher carcass weights of these animals, whereas low-CP males have margins that are comparable to females in both nutritional treatments. The economic margins in this genetic type do not differ across sexes when dietary CP is not limiting (144–153 g CP/kg of feed) [43]. Wang et al. [44] proposed that a moderate reduction in dietary CP reduction (i.e., <2%) would be an economically attractive option to counterbalance cost effectiveness (EUR/kg total N excretion abated) with abatement potential (kg total N excretion/kg body weight gain), but the current results showed that the economic margin from fast-growing Duroc-sired pig males would be more sensible to dietary CP reduction than their female counterparts.

## 5. Conclusions and Future Prospects

This study combines major aspects inherent to a CP reduction strategy counterbalanced by crystalline amino acid supplementation in Duroc X (Landrace X Large White) finishing pigs of both sexes. Overall, it can be stated that the 20 g/kg CP reduction supplemented with amino acids can be efficiently used in heavy fast-growing Duroc sire line genotypes during the finishing phase without a decrease in production parameters. Nonetheless, females had poorer growth performances than males, whereas N efficiency was impaired when females had no limiting dietary CP. Similarly, regarding carcass and meat traits, the strategy may be used efficiently in such animals without leading to major impairments in carcass or meat traits, regardless of the sex. Finally, the economic analysis under the four studied scenarios point to the economic feasibility of all combinations of high- and low-CP feed and carcass prices, with sex being the only factor determining differences in margins per pig. Based on the previously mentioned aspects, it is clear that the reduced CP feeding strategy is feasible and of interest in the framework of pig production. Indeed, its implementation seems furthermore to be particularly beneficial from an environmental sustainability point of view as the amounts of excreted Nitrogen are decreased with additional improvements in N efficiency.

The conducted study rendered very interesting conclusions about the effectiveness of the strategy under commercial pork production conditions with a particular emphasis on growth, carcass and meat traits, in addition to the economic analysis. However, several pertinent aspects could nonetheless be added in future studies. A first suggestion could be to add a sensory analysis to complement meat quality traits. Under the conditions this trial was conducted, we could not perform such analysis. However, it would ultimately reveal if the pork meat from male and female pigs fed the reduced CP diet would have the same acceptance as pork from animals in the control groups. Another important follow-up study may be related to the physiological differences between males and females fed the two different diets. Finally, it would also be very interesting to study the effectiveness of this strategy under different environmental conditions, particularly with high temperature and relative humidity.

## Figures and Tables

**Table 1:** Experimental diets (ingredients, analyzed amino acid composition and calculated nutrients, as-fed basis).

Ingredients (%)	Control CP	Low-CP
Maize	50.00	50.00
Barley 9.6% CP	7.31	16.37
Wheat 10.2% CP	13.00	8.73
Wheat middling	6.00	8.00
Soyabean meal 44% CP	10.22	4.00
Sunflower 36% CP	7.00	5.25
Lard	2.00	2.00
Soybean oil + lecithin	0.70	0.73
Calcium carbonate	1.08	1.28
Monocalcium phosphate	0.25	0.30
Sodium chloride	0.50	0.54
Formic acid	0.10	0.10
Vitamin-mineral premix ^1^	0.20	0.20
Mycotoxin adsorbent	0.10	0.10
Sodium bicarbonate	-	0.16
L-Lysine sulphate 70%	0.93	1.20
Methionine hydroxy analogue	0.17	0.25
L-Tryptophan	0.04	0.07
L-Threonine	0.23	0.33
L-Valine	0.10	0.21
L-Isoleucine	-	0.13
**Analyzed amino acids**		
Crude Protein (%)	14.30	12.20
Total Lys (%)	0.96	0.95
Total Met (%)	0.26	0.21
Total Thr (%)	0.74	0.73
Total Trp (%)	0.21	0.21
Total Val (%)	0.71	0.69
Total Ile (%)	0.58	0.55
Total Arg (%)	0.96	0.72
Total His (%)	0.40	0.32
Total Leu (%)	1.13	0.94
Total Phe (%)	0.70	0.57
**Calculated nutrients**		
DM (%)	86.9	87.0
Net energy for growing pigs (MJ/kg)	10.2	10.2
CP (%)	14.6	12.5
SID Lys (%)	0.87	0.87
SID Met (%)	0.24	0.19
SID Met+Cys (%)	0.47	0.39
SID Thr (%)	0.65	0.65
SID Trp (%)	0.18	0.19
SID Val (%)	0.62	0.61
SID Ile (%)	0.50	0.49
Starch (%)	45.7	48.3
Crude Fiber (%)	4.20	4.10
Ether extract (%)	4.8	4.80
g SID Lys/Mcal NE	3.55	3.55

^1^ Vitamin, mineral and enzyme premix (per kg of complete diet): 6 IU Vitamin A; 1.5 IU Vitamin D3; 15 mg α-tocopherol; 1 mg Vitamin B2; 3 mg Vitamin B2; 1.5 mg Vitamin B6; 0.02 mg Vitamin B12; 0.1 mg folic acid; 20 mg nicotinic acid; 10 mg pantothenic acid; 0.1 biotin; 1 g choline chloride; 94 mg Zn (ZnO); 40 mg Mn (MnO);100 mg Fe (FeCO_3_); 19 mg Cu (CuSO4·5H_2_O); 0.34 mg Se (Na_2_O_3_Se); 50 mg BHT; 0.34 mg I (KI); 750 FYT 6-phytase (Quantum Blue, ABVista, UK; *E. coli*-sourced). DM—dry matter, CP—crude protein, SID—standard ileal digestibility, NE—net energy.

**Table 2:** Effect of low-CP diet with amino acid supplementation on entire male and female pig growth performances.

	Diet-Sex				SEM	*p*-Value		
	Low-CP Female	Low-CP Male	Control-CP Female	Control-CP Male		Diet	Sex	Diet × Sex
BW d0 (kg)	67.2	68.1	67.7	68.7	0.81	0.524	0.257	0.973
BW d21 (kg)	89.8 ^b^	94.4 ^ab^	91.3 ^b^	96.5 ^a^	1.26	0.168	0.001	0.831
BW d42 (kg)	111.6 ^b^	119.6 ^a^	113.3 ^b^	121.6 ^a^	1.39	0.200	<0.0001	0.921
ADG d0–d21 (g/day)	1.076 ^c^	1.252 ^ab^	1.126 ^bc^	1.325 ^a^	35.1	0.099	<0.0001	0.744
ADG d21–d42 (g/day)	1.037 ^b^	1.197 ^a^	1.045 ^b^	1.193 ^a^	40.7	0.965	0.002	0.879
ADG d0–d42 (g/day)	1.056 ^b^	1.225 ^a^	1.085 ^b^	1.259 ^a^	24.5	0.216	<0.0001	0.914
ADFI d0–d21 (g/day)	2.814	2.844	2.780	2.810	78.9	0.670	0.707	0.997
ADFI d21–d42 (g/day)	3.102	3.190	3.086	3.155	80.3	0.756	0.343	0.907
ADFI d0–d42 (g/day)	2.958	3.017	2.933	2.983	72.1	0.685	0.462	0.950
FCR d0–d21	2.62 ^a^	2.28 ^b^	2.47 ^a^	2.12 ^b^	0.040	0.002	<0.0001	0.953
FCR d21–d42	3.03 ^a^	2.67 ^b^	2.96 ^a^	2.65 ^b^	0.107	0.678	0.006	0.801
FCR d0–42	2.80 ^a^	2.46 ^b^	2.70 ^a^	2.37 ^b^	0.042	0.037	<0.0001	0.948

BW—body weight; ADG—average daily gain; ADFI—average daily feed intake; FCR—feed conversion ratio; d0—day 0; d21—day 21; d42—day 42; ^a–c^ Rows with different superscripts indicate significant differences (*p* < 0.05).

**Table 3:** Effect of low-CP diet with amino acid supplementation on entire male and female pig water intake and water:feed ratio.

	Diet-Sex				SEM	*p*-Value		
	Low-CP Female	Low-CP Male	Control-CP Female	Control-CP Male		Diet	Sex	Diet × Sex
Water use (L/pig/day) d0–d21	7.8	7.1	6.6	9.3	1.24	0.679	0.432	0.189
Water use (L/pig/day) d21–d42	10.3	9.0	9.6	10.4	1.22	0.778	0.851	0.423
Water use (L/pig/day) d0–d42	9.0	8.1	8.1	9.9	1.16	0.712	0.745	0.262
Water:feed ratio (L/kg) d0–d21	2.77	2.48	2.39	3.02	0.324	0.812	0.611	0.176
Water:feed ratio (L/kg) d21–d42	3.33	2.82	2.79	3.29	0.330	0.915	0.989	0.145
Water:feed ratio (L/kg) d0–d42	3.05	2.65	2.59	3.15	0.317	0.948	0.800	0.147

**Table 4:** Effect of low-CP diet with amino acid supplementation on entire male and female pig Nitrogen balance and fecal consistency.

	Diet-Sex				SEM	*p*-Value		
	Low-CP Female	Low-CP Male	Control-CP Female	Control-CP Male		Diet	Sex	Diet × Sex
N consumed (g/day)	58.2 ^b^	59.4 ^b^	68.0 ^a^	69.2 ^a^	1.51	<0.001	0.455	0.998
N excreted (g/day)	32.4 ^b^	27.8 ^c^	41.5 ^a^	36.8 ^b^	1.12	<0.001	<0.001	0.936
N retained (g/day)	25.9 ^b^	31.6 ^a^	26.6 ^b^	32.4 ^a^	0.62	0.220	<0.001	0.890
N efficiency (N retained/N consumed)	0.45 ^b^	0.53 ^a^	0.39 ^c^	0.47 ^b^	0.007	<0.001	<0.001	0.536
Fecal DM content d21 (%)	26.9	25.9	27.3	26.9	0.54	0.185	0.210	0.608
Fecal DM content d42 (%)	28.6 ^a^	25.6 ^b^	28.7 ^a^	28.2 ^a^	0.78	0.100	0.041	0.126

DM—dry matter; d21—day 21; d42—day 42; ^a–c^ Rows with different superscripts indicate significant differences (*p* < 0.05).

**Table 5:** Effect of low-CP diet with amino acid supplementation on entire male and female pig carcass traits.

	Diet-Sex				SEM	*p*-Value		
	Low-CP Female	Low-CP Male	Control-CP Female	Control-CP Male		Diet	Sex	Diet × Sex
Carcass weight (kg)	84.5 ^b^	89.9 ^ab^	85.7 ^b^	91.2 ^a^	1.48	0.420	<0.001	0.969
Carcass yield ^1^ (%)	75.8 ^a^	75.1 ^b^	75.6 ^a^	75.3 ^b^	0.22	0.887	0.013	0.416
Lean content (%)	60.9	60.2	60.4	60.0	0.41	0.420	0.192	0.754
Subcutaneous fat thickness of loin (mm)	16.1 ^b^	17.3 ^a^	16.5 ^b^	17.8 ^a^	0.49	0.354	0.011	0.985
Minimal subcutaneous fat thickness of ham (mm)	12.3	12.0	12.7	12.5	0.50	0.377	0.611	0.995
Superior carcasses (>60% lean content) (%)	82.1 ^a^	46.7 ^b^	44.8 ^b^	44.4 ^b^	-	0.004	-	-

^a,b^ Rows with different superscripts indicate significant differences (*p* < 0.05); ^1^ Final body weight was recorded 24 h before the slaughter, and a subsequent fasting period of 18 h was applied.

**Table 6:** Effect of low-CP diet with amino acid supplementation on entire male and female meat traits.

	Diet-Sex				SEM	*p*-Value		
	Low-CP Female	Low-CP Male	Control-CP Female	Control-CP Male		Diet	Sex	Diet × Sex
pH at carcass cutting ^1^	5.28	5.26	5.25	5.24	0.03	0.32	0.56	0.83
Thawing losses (%)	20.00	20.30	19.77	20.72	1.301	0.95	0.65	0.81
Color traits ^1^								
Lightness (L*)	55.91	56.29	54.46	54.29	0.854	0.07	0.91	0.76
Redness (a*)	3.06	3.08	2.84	3.19	0.424	0.90	0.68	0.72
Yellowness (b*)	10.64	10.34	9.98	9.48	0.373	0.07	0.31	0.79
Hue angle	74.10	74.00	74.10	71.50	1.92	0.53	0.50	0.54
Chroma	11.08	10.85	10.39	10.02	0.44	0.11	0.52	0.89
Chemical composition ^2^								
Ash (%)	1.85	1.81	1.84	1.82	0.030	0.96	0.35	0.70
Moisture (%)	71.95	71.53	72.70	72.46	0.379	0.05	0.41	0.82
Protein (%)	25.68	25.91	26.38	25.91	0.200	0.11	0.57	0.11
Fat (%)	4.17 ^a^	4.33 ^a^	2.92 ^b^	3.55 ^ab^	0.395	0.02	0.34	0.57
Saturated fat (%)	1.34 ^a^	1.34 ^a^	0.81 ^b^	1.00 ^ab^	0.164	0.02	0.59	0.60
Collagen (%)	0.89	0.96	0.86	0.90	0.033	0.25	0.11	0.62
Sensory traits ^2^								
Hardness (N)	29.7	31.9	39.9	35.0	5.51	0.26	0.82	0.54
Cohesiveness	0.30	0.29	0.27	0.28	0.011	0.20	0.86	0.35
Springiness	4.43	4.90	3.47	3.80	0.696	0.17	0.59	0.93

^a,b^ Lines with different superscripts indicate significant differences (*p* < 0.05); ^1^ Parameter determined for all animals in the study; ^2^ Parameters determined for one animal per pen using Texture Profile Analysis.

**Table 7:** Economic assessment of the effect of low-CP diet with amino acid supplementation on entire male and female pigs.

	Diet-Sex				SEM	*p*-Value		
	Low-CP Female	Low-CP Male	Control-CP Female	Control-CP Male		Diet	Sex	Diet × Sex
Low feed and carcass prices scenario (2020) ^1^								
Feed cost (EUR/pig)	112.05	112.69	112.52	113.07	0.788	0.599	0.461	0.956
Carcass income (EUR/pig)	142.04 ^c^	150.00 ^ab^	143.99 ^bc^	152.64 ^a^	1.647	0.182	<0.001	0.833
Margin-over-feed-cost (EUR/pig)	29.99 ^b^	37.30 ^a^	31.47 ^b^	39.57 ^a^	1.249	0.153	<0.001	0.754
High feed and carcass prices scenario (2022) ^2^								
Feed cost (EUR/pig)	133.17	134.23	135.92	136.87	1.325	0.059	0.459	0.967
Carcass income (EUR/pig)	177.09 ^b^	184.70 ^ab^	179.39 ^ab^	188.10 ^a^	3.281	0.397	0.024	0.869
Margin-over-feed-cost (EUR/pig)	43.91 ^b^	50.46 ^a^	43.47 ^b^	51.23 ^a^	3.309	0.962	0.046	0.857
High feed and low carcass prices								
Margin-over-feed-cost (EUR/pig)	8.87 ^b^	15.76 ^a^	8.06 ^b^	15.77 ^a^	1.223	0.749	<0.001	0.742
Low feed and high carcass prices								
Margin-over-feed-cost (EUR/pig)	65.03 ^b^	72.00 ^ab^	66.87 ^ab^	75.03 ^a^	3.230	0.463	0.032	0.856

^a–c^ Rows with different superscripts indicate significant differences (*p* < 0.05); ^1^ Feed prices for the most favorable scenario (2020) were EUR 0.258/kg and EUR 0.264/kg (+EUR 6/t) in control and low-CP feeds, respectively. The feed price of the control diet was set according to mean values of finishing feed in Spain throughout 2020 (MAPA, 2023); ^2^ Feed prices for the least favorable scenario (2022) were EUR 0.428/kg and EUR 0.454/kg (+EUR 26/t) in control and low-CP feeds, respectively. The feed price of the control diet was set according to mean values of finishing feed in Spain throughout 2022 (MAPA, 2023). In all cases, feed cost was calculated by multiplying feed price (EUR/kg) by amount of feed consumed during the 42 days of the experiment (in kg/pig), plus a fixed partial feed cost of EUR 80/pig from 6 to 70 kg of BW (start of the experiment).

## Data Availability

Access to original data will be granted by the original author upon reasonable request.
